# Bridging the Gap: A Case Study of Tailored Support for Students with Social, Emotional, and Behavioral Needs During the Transition to High School

**DOI:** 10.3390/bs16060984

**Published:** 2026-06-12

**Authors:** María Reina Santiago-Rosario, Sarah Fairbanks Falcon, Sean C. Austin, Joseph F. T. Nese, Maeghan M. Sullivan, Tony Daza, T. Elyse Calhoun, Haley Cerdan, Rhonda N. T. Nese

**Affiliations:** 1 College of Education, University of Oregon, Eugene, OR 97403, USA; sfalcon@uoregon.edu (S.F.F.); seana@uoregon.edu (S.C.A.); jnese@uoregon.edu (J.F.T.N.); daza@uwp.edu (T.D.); tcalhou2@uoregon.edu (T.E.C.); hbrown6@uoregon.edu (H.C.); rnese@uoregon.edu (R.N.T.N.); 2 School of Education, Lewis & Clark College, Portland, OR 97219, USA

**Keywords:** high school, middle school, transition, student-centered, parent coaching

## Abstract

Students with disabilities, particularly those needing additional support or intervention to manage emotions and behaviors, build healthy relationships, and navigate social and academic demands, face heightened risks of high school pushout that can be traced back to their transition into high school. Project Elevate (PE) is a multi-component intervention that strategically invests in early coordinated student, family, and school supports to prevent barriers associated with high school pushout, such as a lack of continuity of effective services across school sites. This mixed-methods pilot study examined the implementation of PE with three 8th-grade students and their parents during their last term in middle school. This study includes quantitative pre–post descriptive analyses of multi-informant reports of students’ social, emotional, and behavioral skills, as well as descriptive analyses of weekly teacher- and parent-reported behavior and student attendance. Qualitative analysis using the Framework Method was applied to student and parent interviews and open-ended responses on a satisfaction questionnaire to understand their experience receiving PE support. Session case notes were also used as contextual data to describe implementation processes and contextualize findings. Results indicated improvements in student attendance and reductions in home-based behavioral concerns, with mixed findings across school-based outcomes. Students and parents reported high satisfaction with the intervention, highlighting the value of individualized support, goal setting, and strengthened communication with schools. Findings from this intervention development pilot study provide preliminary evidence regarding the implementation and perceived value of PE. Results also highlight the importance of culturally responsive, relationship-centered practices that affirm student strengths and support access to educational opportunities. Further investigation of PE in larger studies is warranted.

## 1. Introduction

In the United States, the transition from middle (typically sixth through eighth grade) to high school (ninth through twelfth grade) brings numerous academic and social challenges. Transitioning to high school is difficult for many students as they adapt to a larger campus, more challenging coursework, and expectations of greater independence, coupled with the absence of established relationships and support from middle school (Lopez et al., 2020; McFarland et al., 2020). Even high-achieving eighth graders need extra support to thrive in high school (Baharav & Sipes, 2020; McMahon & Sembiante, 2020). This is critical, as nineth grade course performance and attendance are among the strongest predictors of graduation and dropout (Kearney et al., 2023). Although 87% of first-time ninth graders earn a diploma in four years, this rate drops to 71% for students with disabilities (Irwin et al., 2024). Among them, those who qualify for special education services primarily under emotional and behavioral disorders (EBDs) and Other Health Impairments face the highest dropout rates (U.S. Department of Education, 2024). Despite this, research on effective middle-to-high school transitions to support individual students with disabilities is limited. Research has primarily focused on universal systems and class-wide interventions rather than more individualized support, especially for those with disabilities (Cavendish & Connor, 2018; Lindstrom & Beno, 2020; Šiška et al., 2024). This is often magnified by a lack of implementation support. Coaches, case managers, and transition support teams at the secondary level are often over-burdened with high caseloads or too under-trained to assist effectively (Buchanan & Clark, 2017; Nuske et al., 2019; Strnadová et al., 2023). However, when implemented with fidelity, student and parent coaching can positively impact outcomes by modeling strategies directly (Fosco et al., 2013; Sinclair et al., 2005). An additional implementation support ingredient that can increase such coaching gains is case management. Beyond siloed coaching, case management builds on coaching success and provides an infrastructure to coordinate and triage between youth, school, and family (Buchanan et al., 2016a). These building blocks and bridging mechanisms (teaming, coaching, and coordination) are important considerations during the complex high school transition period. This study addresses the gap by piloting a comprehensive, multi-component model developed with input from educators, parents, and students with disabilities to support the middle-to-high school transition. Project Elevate (PE) was designed to complement existing school-based behavioral health and special education support with consistent coaching and coordination support across students, families, and school teams during the middle-to-high school transition. This study presents preliminary findings of the impact of PE for three eighth graders receiving special education services.

### 1.1. Students Facing the Greatest Transition Challenges

Several factors shape how students adjust to high school. Successful high school graduates often cite strong relationships with teachers, family, and peers, along with the ability to handle increased academic demands (Benner et al., 2017; Longobardi et al., 2016, 2019; Maxwell et al., 2017). Students with strong transition-relevant adaptive skills such as goal setting, task initiation, understanding consequences, and maintaining supportive friendships tend to experience more successful transitions from middle to high school (Lessard & Juvonen, 2022; Rowe et al., 2015; Roybal et al., 2014). These students often have strong social, emotional, and behavior (SEB) skills or the ability to integrate social skills, emotional control, and behavioral habits to function adaptively in everyday settings (Soto et al., 2022). Conversely, students with SEB challenges have difficulty regulating their emotions and using social skills, leading to behavior that disrupts academic and social success (Soto et al., 2022). These same students often report low self-determination, self-advocacy, and emotional regulation (Cameto et al., 2004; Dishion et al., 1991; Okano et al., 2020), which can hinder both academic and personal growth, as these skills are crucial for positive educational and post-secondary opportunities (Karvonen et al., 2004). Students with SEB challenges also report feeling disconnected from school (A. Y. Liu et al., 2018), perceive a worsening school climate in high school (Gage et al., 2021), have a higher incidence of disability (Soto et al., 2022), and face higher rates of mental health issues and peer victimization (La Salle et al., 2018). Thus, individualized interventions designed in collaboration with the youth’s support systems involved are essential to foster their academic and SEB growth during this critical transition.

### 1.2. Promising Evidence-Based Practices

Understanding and addressing the SEB assets and needs of students with disabilities during the middle-to-high school transition can significantly improve short- and long-term outcomes (Strnadová et al., 2023). Although few interventions are designed specifically to support students with SEB challenges during this transition period, prior research has identified some relevant interventions. For example, Check and Connect includes structured mentoring to maintain student engagement during this important high school pivot point (Sinclair et al., 2005). Similarly, the Self-Determined Learning Model of Instruction supports students in developing the self-management skills necessary to navigate changing academic expectations (Shogren et al., 2024). Despite these approaches, a gap remains regarding multi-component models that combine input from students, parents, and educators and coordinated care to support students with acute SEB needs through the shift to high school. To date, these types of support have most often been implemented at the universal or class-wide level or outside the context of coordinated transition planning (McGrath Kato et al., 2018), highlighting the need to integrate and adapt them into targeted transition-focused models.

PE was designed to address this gap by integrating evidence-based components drawn from the literature and from two previously evaluated interventions (Students with Involved Families and Teachers (SWIFT, Buchanan et al., 2016b) and Freshmen Success (Flannery et al., 2020)), while extending these approaches to focus explicitly on the middle-to-high school transition. PE is grounded in an integrative framework combining social and behavior theory and self-determination theory. PE emphasizes that behaviors are learned through modeling and teaching, and that environmental factors influence when and how a behavior is likely to occur (Sugai & Horner, 2009). Therefore, new prosocial behaviors can be taught and reinforced through a systematic plan of teaching and reinforcement, with additional environmental changes. Rather than operating as isolated practices, the six components of PE interact dynamically across a student’s life. Core components include skills coaching, student-led goal setting and progress monitoring, parent involvement, case management, and peer mentorship, all of which have independently demonstrated positive effects for students with SEB needs (Flannery et al., 2020; Newton et al., 2012; Park et al., 2011; Ryan, 1970; Shogren et al., 2024; Taylor et al., 2017). These components start by emphasizing environmental stabilization with parent support and case management, then move towards capacity building in the form of skills coaching, and finally internal motivation and autonomy by harnessing self-determination in the form of goal setting and progress monitoring affecting individual agency and impacting longer-term adaptive behaviors (e.g., study skill routines or adapting communication styles).

**Figure 1:**
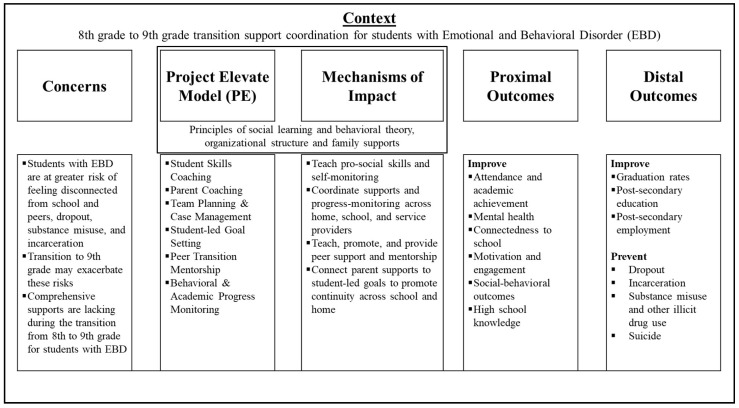
This figure displays Project Elevate Theory of Change, which illustrates the theorized relationships between intervention components, mechanisms of impact, and proximal and distal outcomes within the context of middle-to-high school transition for students with emotional and behavioral disabilities.

Skills coaching supports the development of prosocial, organizational, and self-advocacy skills, which are associated with improved emotional and behavioral regulation, academic success, and peer and teacher relationships (Jennings & DiPrete, 2010; M. P. McCormick et al., 2015; Nese et al., 2021a; Taylor et al., 2017). Student-led goal setting is grounded in goal-setting theory, which posits that conscious goal development influences actions (Ryan, 1970). Students with higher self-efficacy set more challenging goals, use better strategies, and respond more positively to feedback (He et al., 2023; J. Liu et al., 2023). Regular progress monitoring further supports adaptive adjustments to instruction and services during transitional periods (Savas, 2023; Shogren et al., 2024). Parent involvement enhances the effectiveness of behavioral and transition interventions, particularly for students with acute SEB needs, by promoting consistency across home and school contexts (Buchanan et al., 2016b; Lambert et al., 2022; Park et al., 2011). Case management ensures coordination across middle and high school systems, reducing the fragmentation of services and reliance on restrictive placements while supporting collaborative problem-solving among teams (Anaby et al., 2019; Horner et al., 2018; McClain et al., 2024; S. T. McCormick et al., 2021). By integrating these evidence-based elements into a coordinated transition model, PE seeks to provide targeted support during a critical window for youth. This approach remains rare despite evidence that such integration can improve graduation rates and overall educational experiences for students with significant SEB needs (McGrath Kato et al., 2018). Figure 1 shows PE’s theory of change.

### 1.3. Designing Project Elevate: A Systematic Approach for High School Transition

Building on the evidence-based practices described above, PE is a tailored intervention supporting special education students with SEB and academic learning needs as they transition to high school. Grounded in social and behavioral theory, PE emphasizes modeling, teaching, and environmental influences on behavior (Patterson, 2005). Rather than introducing new intervention strategies, PE operationalizes and integrates established evidence-based components within a coordinated transition model focused specifically on the middle-to-high school transition. This approach was designed to operate within existing school structures and emphasizes communication and coordination as mechanisms of change that intentionally sit across home and school systems to support students’ transition to high school. Designed to align with tiered systems of support (e.g., multi-tiered systems of support [MTSS] or positive behavioral intervention and supports [PBIS]), PE provides individualized, coordinated transition support that complements existing Tier 3 academic and SEB interventions rather than introducing parallel structures. The model includes six core practices: student skills coaching, parent coaching, team planning and case management, student-led goal setting, peer mentorship, and progress monitoring.

Each student is paired with a skills coach for weekly one-hour sessions focused on prosocial skill development, problem-solving, and goal setting aligned with their transition from the eighth to nineth grade. A parent coach supports the primary parental figure through weekly sessions, helping them build home systems that reinforce student skills and improve school communication. A transition team, comprising students, parents, coaches, and school staff, meets once or twice per semester to review progress and provide feedback. These team meetings are coordinated by the PE case manager, who serves as the primary coordination lead and acts as the main liaison between PE coaches and the school and the special education case managers. Academic and behavior progress monitoring is tracked using the Parent Daily Report (PDR) and Teacher Daily Report (TDR), along with student and parent goal tracking. Peer mentorship begins once students enter high school, pairing them with older students for guidance and support. In the current pilot, this component was planned but not implemented due to the timing of the study, which concluded during the final term of middle school. Together, these coordinated practices are designed to ensure the continuity of support across home and school.

Although high school preparedness sometimes begins in middle school, existing transition strategies are typically implemented at the universal or class-wide level and are rarely tailored to students at a heightened risk of dropping out (Flannery et al., 2020; Somers & Garcia, 2016). To inform the development of PE with attention to cultural and contextual fit and feasibility within existing school systems, the team examined a district’s existing transition process and gathered input from eighth and nineth graders receiving special education services, their families, and educators (Nese et al., 2021b). Educators expressed concern and historical struggle about graduation rates and emphasized the need to explicitly teach executive functioning (e.g., organizational, breaking down work), self-advocacy, and communication skills. Parents described the isolation their children experienced during this transition and highlighted the need for better information sharing, exposure to the high school setting and logistics (e.g., high school tour/open house, first day orientation, and welcome video), and Individualized Education Program (IEP) support. Ninth graders identified navigating the school building, academic workload and rigor, and making friends as major challenges. Many said they wanted someone to check in with and offer advice. Drawing from their input and prior intervention research, PE was intentionally designed as a targeted, system-coordinated transition intervention for special education students at heightened risk of school disengagement and dropout. PE focuses on strengthening connection, emotional regulation, social, behavioral, and academic skills, and goal-directed behavior during the transition to high school.

### 1.4. Purpose of the Study

Although research supports the need for preventative high school preparedness for students with SEB needs, there is limited evidence describing comprehensive, multi-component transition support for students with SEB challenges moving from middle to high school. This intervention development pilot study documents the implementation of PE during students’ final term of middle school and describes preliminary descriptive outcomes, including student attendance and engagement, social skills, and behavior across home and school. The current study sought to answer the following questions:

1. What descriptive changes are observed in student attendance, engagement, social skills, and behavior during the implementation of PE?
2. How do students with disabilities and their parents perceive the relevance, practicality, and utility of the PE model?

## 2. Materials and Methods

### 2.1. Participants and Settings

Students from two public middle schools (Journey and Voyage) in different school districts in the Pacific Northwest region of the United States participated in this study. Journey Middle School enrolled 446 students, with 17% receiving special education services. Of the total student population, 57% identified as White and 43% identified as a minority race/ethnicity or two or more races. Voyage Middle School had 826 students enrolled, with 13% receiving special education services. Of the total student population, 48% identified as White and 52% of students identified as a minority race/ethnicity or two or more races. The special education case manager at Journey Middle School identified as a White male with multiple years of teaching experience, and the case manager at Voyage Middle School identified as a White female with multiple years of teacher experience. Three students (i.e., Andy, Barry, and Daniel) participated in the study with their primary parental figure (i.e., Helen, Nora, and Diana). All participant names have been changed.

#### 2.1.1. Andy and Helen

Andy, a 14-year-old 8th grader at Journey Middle School, qualified for special education services under Emotional Disturbance. He and his mother, Helen, identified as White and spoke English. Andy lived with Helen after relocating due to his father’s struggles with alcohol. According to Andy, the move provided him with a more stable home environment. At baseline, Helen indicated that Andy showed more consistent behavior regulation at home alongside more variable peer interactions in the community. Consistent with the first Parent Daily Report, his prosocial behavior at home was characterized by following rules and directions and cooperating, alongside unwanted behaviors such as arguing, talking back, and noncompliance. Teacher report at baseline indicated lower social interaction and greater difficulty sustaining engagement at school. Consistent with the first Teacher Daily Report, prosocial behavior at school primarily involved cooperation and participation, whereas unwanted behavior included interrupting, swearing, and not following directions.

#### 2.1.2. Barry and Nora

Barry, a 14-year-old 8th grader at Voyage Middle School, also received special education services under Emotional Disturbance. He and his mother, Nora, identified as Latine. Barry was bilingual in English and Spanish. Nora spoke Spanish and held undocumented status during this study. They lived in a one-bedroom apartment in the Pacific Northwest, along with Nora’s long-term partner. At baseline, Nora indicated that Barry demonstrated strong social engagement at home with variability in how consistently he managed behavior across situations. Consistent with the first Parent Daily Report, his prosocial behavior at home was characterized by helping others, participating, and following directions, alongside unwanted behaviors such as arguing, talking back, and irritability. Teacher report at baseline indicated lower levels of peer and social functioning at school. Consistent with the first Teacher Daily Report, prosocial behavior at school primarily involved cooperation and following directions, whereas unwanted behavior included arguing, talking back, and irritability.

#### 2.1.3. Daniel and Diane

Daniel, a 14-year-old 8th grader at Voyage Middle School, received special education services under Other Health Impairment. He and his mother, Diana, identified as White and spoke English. Daniel lived with his mother Diana and a sibling following her recent divorce from his father. At baseline, Diana indicated that Daniel demonstrated generally consistent behavior at home, alongside less developed peer interactions. Consistent with the first Parent Daily Report, his prosocial behavior at home was variable but included helping others and participating, alongside unwanted behaviors such as anxiety, irritability, and noncompliance. Teacher report at baseline similarly indicated lower social engagement at school. Consistent with the first Teacher Daily Report, prosocial behavior at school primarily involved cooperation, whereas unwanted behavior included anxiety, irritability, and inconsistently following directions.

### 2.2. Research Design

This pilot study used a convergent mixed-method case study design to examine the experiences of students and parents receiving PE support. This mixed-method design reflects collecting qualitative and quantitative data concurrently but separately during the intervention process to understand behavior changes and experiences with PE, with the merging of data occurring after independent analyses during interpretation (Clark et al., 2018). Data integration occurred at the individual case level through a process of comparing quantitative patterns (e.g., attendance, behavior ratings) with qualitative findings from analyzed interviews and open-ended survey data, and drawing on session case notes as acontextual information, to examine areas of agreement, disagreement, or complement. We combined data types to better understand observed changes during the intervention and contextualize them with reported experiences. Quantitative data included student self-report, as well as parent and teacher reports on students’ SEB outcomes, along with a satisfaction questionnaire completed by parents and students. To understand individual experiences and contexts, qualitative data included exit interviews and open-ended satisfaction questionnaire responses (formally analyzed using the Framework Method), whereas session case notes were used to contextualize findings and describe implementation processes across cases. Thus, qualitative data were used to contextualize observed patterns in quantitative outcomes. Consistent with the goals of a descriptive pilot study and acknowledging available resources, the sample size was selected to allow for qualitative exploration of participant experiences while also generating descriptive quantitative data to inform future refinement and procedures. Findings are presented using a narrative approach that wove together quantitative and qualitative evidence for each participant to support case-level interpretation.

### 2.3. Measures

#### 2.3.1. Session Case Notes

Session case notes provided structured documentation to track delivery of support, progress, and engagement during coaching sessions with students and parents. Notes included the date, subjective impressions to capture interpersonal dynamics or self-report, an objective section detailing actions, behaviors, and topics, an assessment subsection summarizing qualitative and quantitative data collected, and a next steps section outlining goals, strategies, or recommendations to guide future sessions. Case notes were recorded by the PE case manager during phone call check-ins immediately following each coaching session.

#### 2.3.2. SEB Outcomes

Student data were collected using multiple measures across parents, teachers, and students. Parents completed the Parent Daily Report (PDR) weekly via a 5 min in-person conversation during sessions or phone interviews. The PDR includes 54 common behaviors (34 undesirable and 17 prosocial), with ratings on occurrence and stress level of undesirable behaviors over the past 24 h. The PDR has been used in numerous treatment outcome studies (Hurlburt et al., 2010; Price et al., 2012) and has high inter-rater reliability (r = 0.98) and temporal stability (r = 0.82). Parents also completed the Home and Community Social Behavior Scale (HCSBS) pre- and post-intervention to assess social skills strengths and deficits. The HCBS has acceptable internal consistency (α = 0.94 to 0.98), test–retest reliability (r = 0.60 to 0.91), and inter-rater reliability (r = 0.53 to 0.86).

Teachers completed the Teacher Daily Report (TDR) weekly via a 5 min in-person conversation or phone interview. The TDR mirrors the PDR format with 63 behaviors (42 undesirable and 21 prosocial) capturing occurrence in the past 24 h. They also completed the Walker–McConnell Scale (WMS; Walker et al., 1991) pre- and post-intervention to assess teacher–student relationships and school adjustment. The WMS shows strong test–retest reliability (r = 0.88 to 0.97) and internal consistency (α = 0.90), with adequate inter-rater reliability (r = 0.53 to 0.77).

Students completed the Student Engagement Instrument (SEI; Appleton et al., 2006), a 33-item measure of cognitive and affective engagement. The SEI is divided into six subscales (i.e., future goals and aspirations, control and relevance of schoolwork, extrinsic motivation, family support for learning, peer support for learning, and teacher–student relationships), has internal consistency ranging from 0.72 to 0.88, and predicts on-time graduation and dropout risk.

#### 2.3.3. Student-Led Goal Setting

Each student identified a primary goal to work on with their PE skills coach. Across participants, students elected to work on improving their attendance. Attendance data were organized into three predefined phases. The baseline phase included available data from that term collected prior to the start of coaching. The remaining observation period was divided into two phases (middle and final), with each phase representing an equal number of weeks following the initiation of coaching.

#### 2.3.4. Social Validity

Social validity was assessed using the Client Satisfaction Questionnaire (CSQ-8) (Larsen et al., 1979), completed by students and parents. This 8-item, 4-point scale (score range 8–32) measure had content validity established through expert review and factor analysis. As this measure was designed to gather input from clients or parents, minor word changes were made to ensure that students could also respond. Semi-structured exit interviews with students and parents further explored perceptions of PE components, including helpful components, areas for improvement, and how PE helped with high school preparedness.

### 2.4. Procedure

#### 2.4.1. Recruitment, Consent, and Activities Timeline

After approval from the university’s Institutional Review Board (Protocol #10252019.036, 29 November 2022), we identified school districts with multiple middle schools serving 2–5 students (consistent with national averages) who qualified for special education services primarily under the category of EBD. We met with district-level student services directors to describe the pilot study, identify the number of 8th grade students meeting that criteria, and discuss next steps for contacting middle school administrators. Separate meetings across districts were then held between middle school principals and multiple 8th graders who qualified for special education, primarily under EBD, to describe the study procedures and the process for randomized assignment to intervention or waitlist-control conditions. At that time, project staff discussed recruitment procedures in greater detail with school administrators. Administrators across schools determined that the most appropriate approach for inviting students and their families was through the special education teachers who served as case managers responsible for overseeing the students’ IEP. Special education case managers were identified and invited to participate. Project staff met with these case managers to explain the study, review and obtain informed consent, and initiate the recruitment process of student–parent dyads. Between October and December 2022, special education case managers distributed invitation letters outlining project details, followed by phone calls to address questions and connect interested families with the research team. The special education case managers shared contact information for families who expressed interest, allowing project staff to coordinate meetings to review consent and assent procedures and discuss study activities with both students and their parents. No data from control schools are reported in this study, because families meeting criteria in control schools did not express interest in participating in data collection activities. Consent and assent discussions were conducted by research staff and emphasized that participation was voluntary, that families could decline or withdraw at any time without penalty, and that participation would not affect students’ special education services or school standing. Given involvement of students with disabilities, additional care was taken to ensure the assent procedures were developmentally appropriate and that students understood the purpose of the study and their right to decline participation. Parents and students who agreed to participate completed the consent and assent discussion in January and February 2023 and scheduled initial coaching sessions with the PE case manager. Parent–student dyads were consented and onboarded through this initial recruitment effort. Due to the small pool of students receiving services under EBD as the primary learning disability category, a second wave of recruitment was initiated to include families of students receiving services under other learning disabilities. Specifically, the special education case managers identified students who observed an increase in unwanted behavior and repeated the same process. One additional dyad consented. Recruitment timelines resulted in staggered intervention start dates, with Andy and Barry beginning in February and Daniel beginning in March.

#### 2.4.2. Coaching Participation and Data Collection Overview

Students and parents participated in coaching sessions with their respective coaches. The duration of skill coaching sessions with students ranged between 15 and 60 min; the parent coaching session duration ranged between 60 and 90 min. All participants completed pre- and post-intervention surveys during the weeks of the first and final coaching sessions, along with exit interviews during the final week of intervention.

#### 2.4.3. Structure of Skill Coaching and Parent Coaching Sessions

The structure of skill coaching sessions took the following form: an initial check-in regarding participants’ week and completing any assessments due; engagement in a relationship-building activity; discussion of progress toward a self-selected goal; and identification of strategies to support desired changes and next steps. Similarly, parent coaching sessions began with a weekly check-in and completion of the weekly PDR and other measures when due, followed by a discussion of goals related to supporting their adolescent and/or personal self-care, collaborative problem solving, and identification of strategies to support next steps.

The first month of the intervention focused on building relationships between students and skill coaches and between parents and parent coaches. Student skills coaches used the initial three to four sessions for introductions, expectation setting, and activities designed to explore students’ backgrounds, strengths, interests, and needs. These activities included the Values Card Sort (Miller et al., 2001), a reinforcer assessment to identify incentives, and a goal preference assessment, which supported initiation of SMART goal setting by sessions four or five. Parent coaching followed a similar structure during the first three sessions, incorporating introductions, expectation setting, and values identification using the Values Card Sort (Miller et al., 2001). Parent coaches collaborated with parents to establish SMART goals for supporting both themselves and their adolescents, with subsequent sessions focused on skill development aligned with those goals. As coaching progressed, final sessions focused on synthesizing skills and preparing students to organize materials, reflect on progress toward goals, and practice articulating goals and support needs in anticipation of an end-of-year transition meeting. The Supplemental Tables S1 and S2 provide summaries of student coaching and parent coaching session details.

Following completion of coaching sessions, each student participated in an end-of-year transition meeting designed to consolidate progress, reflect on goal attainment, and identify next steps for the transition to high school. These meetings were planned and organized by the PE and special education case managers and led by the student with support from their PE coach. All relevant school and family members were invited to attend. The meetings provided an opportunity for students to present goals, report on strategies used and growth, and discuss anticipated support and accommodation needed in high school.

#### 2.4.4. Supervision and Coordination Structures Supporting Services Provided

To support consistency in coaching processes across student–parent dyads, structured supervision, documentation, and coordination routines were embedded and used to monitor implementation processes across sessions. Coaches participated in weekly phone calls with the PE case manager to debrief about what happened during the session. This time served as an opportunity to share activities that took place during the session and record events through case notes. PE case managers also used this opportunity to identify discussion topics for group supervision next time. Prior to each group supervision meeting, PE case managers and the core research team met to review logistical aspects of the project and identify key updates to communicate with coaches, supporting consistency in messaging and planned activities across sites. The PE case manager facilitated these meetings, which followed a structured and recurring agenda guided by meeting minutes projected during the session. Meetings typically began with a brief check-in and announcements, followed by project logistics and then structured case reviews and updates. During case reviews, coaches reported on sessions using a consistent format, including activities completed, participant engagement, relational dynamics, and goal-related data updates. Previous meeting notes were used to guide follow-up discussion and ensure continuity across sessions. Skills and parent coaches reported sequentially, allowing for cross-case comparison and alignment of coaching approaches, with additional input provided by other team members as needed. This structured supervision routine provided ongoing opportunities to review session content, align coaching practices across roles, and ensure consistency between session delivery and planned PE components. A total of fifteen and fourteen 60 min group supervision meetings were held for coaches serving dyads attending Journey and Voyage Midde Schools, respectively. Additionally, skills and parent coaches participated in six monthly didactic training sessions designed to support consistency in coaching approaches across staff and to build core competencies central to the PE model. These trainings covered topics and skills like defining the coach’s role, expectations, and responsibilities; session structure and pacing; and suggested activities aligned with PE goals. Training content focused on developing skills in coaching, relationship building, use and development of SMART goals, progress monitoring, and strategies for supporting student and family engagement across sessions. These trainings were used to promote consistency in coaching practices and topics discussed in session across stages of the intervention. Lastly, weekly communication between the PE case manager and the school-based special education case manager further supported alignment across students, families, and schools. The PE case managers engaged in weekly phone calls with the special education case managers (*M* = 10 min, range = 5–20 min) to exchange updates, support coordination, and complete the TDR and other required measures.

### 2.5. Data Analysis

#### 2.5.1. SEB Outcomes

Data from the student-identified goal, PDR, TDR, HCSBS, WMS, and SEI were analyzed descriptively. All figures were created in R (version 4.6.1; R Core Team, 2024) with the following packages: ggthemes (version 5.1.0; Arnold, 2024), janitor (version 2.2.0; Firke, 2023), here (version 1.0.1; Müller, 2020), readxl (version 1.4.3; Wickham & Bryan, 2023), and tidyverse (version 1.3.0; Wickham et al., 2019).

#### 2.5.2. Social Validity

We used the Framework Method (Gale et al., 2013) to manage, summarize, and analyze responses from exit interviews and open-ended CSQ-8 items. Transcripts were generated using Otter.ai and reviewed for accuracy by a member of the research team. Familiarization with the data began with repeated review of interview transcripts and questionnaire responses. Two team members conducted line-by-line coding of transcripts and open-ended responses independently. Because the interviews were brief and structured around specific components of PE, only segments relevant to theory-driven PE mechanisms (e.g., skills coaching, parent coaching, goal setting, and progress monitoring) and perceptions of high school preparedness were coded and analyzed. In contrast to Gale et al.’s (2013) recommendation to iteratively develop a detailed working analytical framework, the structured nature of the interview prompts and the concise, descriptive responses meant that participant data mapped closely onto the predefined PE components. As a result, the analytic framework was derived directly from these domains rather than through multiple rounds of code refinement. This approach is consistent with the flexibility described by Gale et al. (2013) for studies with focused, deductive aims and highly structured data. Following coding, data were summarized into a Framework Method matrix, with each dyad represented in rows and analytic domains represented in columns. Each cell contained condensed summaries of relevant coded data and quotes. Matrix charting was completed independently and then reviewed collaboratively by the research team. Although Gale et al. (2013) describe applying a finalized analytical framework to subsequent transcripts, the simplicity and consistency of participant responses meant that the initial domain structure remained sufficient throughout analysis. Team discussions ensured shared interpretation and consistent indexing across transcripts, aligning with the intent of Stage 5 while adapting the process to the characteristics of the dataset. Discrepancies in coding or interpretations were discussed and resolved through consensus. Analytic interpretation involved examining patterns within and across participants within each domain to identify similarities, differences, and illustrative examples of participant experiences. Findings are presented as domain-based summaries supported by participant quotations, reflecting how students and parents described their experiences with specific components of PE and their perceptions of high school preparedness.

### 2.6. Positionality Statement

This mixed-methods descriptive pilot study was guided by participatory–pragmatic orientations (Clark et al., 2018). From a participatory perspective, the research was oriented toward addressing inequities experienced by students with disabilities in relation to high school preparedness, with particular attention paid to empowerment and voice. From a pragmatic orientation, methodological decisions were driven by the research questions and the intended consequences of the work, prioritizing approaches that could inform practice and support meaningful change. Analyses were led by three authors, with analytic decisions developed through collaborative team discussions. All authors contributed to the broader study through model conceptualization, coach training and supervision, coordination with school partners, and engagement with students and parents. These roles positioned the research team in close relationship with the intervention context, requiring ongoing reflexivity about how researchers’ experiences and assumptions shaped data interpretation. Reflection was supported through weekly supervision meetings in which coaches engaged in collective reflection, problem-solving, and case planning. These discussions functioned as analytic spaces to surface multiple perspectives and attend meaning making. The research team included researchers with diverse racial, linguistic, gender, neurodiversity, and educational backgrounds, which informed a deliberate emphasis on centering student and parent perspectives and interpreting findings within their social and institutional contexts. The next section presents results, emphasizing the importance of context and understanding student and parent experiences.

## 3. Results

This section reports findings organized by the two research questions guiding this descriptive pilot study. Research question 1 focuses on the descriptive changes in student attendance and engagement, social skills, and behavior documented over the course of PE implementation. Information gathered from parent coaching sessions was incorporated under this question for additional context, providing insights into routine-setting, communication patterns, and family-level factors present at the time. Research question 2 addresses student and parent perceptions of the relevance, practicality, and utility of the PE model. Under this question, exit interviews and open-ended questions from the CSQ-8 were treated as primary data sources.

### 3.1. What Descriptive Changes Are Observed in Student Engagement, Social Skills, and Behavior During PE Implementation?

#### 3.1.1. Student Participation Context and Self-Selected Goal

Three student–parent dyads participated in PE during the spring semester: Andy and Helen, Barry and Nora, and Daniel and Diana. Students varied in instructional placement, attendance patterns, behavioral presentation, and length of participation. Across cases, skills coaching emphasized rapport-building, goal setting, and progress monitoring with a focus on attendance, engagement, and social–behavioral functioning. Parent coaching sessions occurred separately and focused on routines, reinforcement strategies, communication with schools, and caregiver self-reflection and care in relation to student goals.

##### Andy

Andy began PE in mid-February and completed 14 weekly in-person coaching sessions (30–60 min each) in school through early June. Coaching sessions focused on rapport-building, addressing attendance, practicing self-regulation, and strategizing for success in school. He attended core classes in special education and one general education elective and had a modified schedule due to difficulties with attendance, lunchroom behavior, and peer interactions. Andy struggled with social cues and emotional regulation and experienced several behavioral incidents, including a suspension. Andy identified his top values as “taking care of my family”, “being kind”, “being a good student and peer”, “not giving up”, and “being physically active.” He identified one-on-one times with adults, playing games, and music as positive reinforcers. With coaching support, Andy set SMART goals related to school attendance and to stay in school every day until spring break. He identified sleep and wake-up routines as areas of focus for arriving at school on time. Given his mother’s work schedule, Andy took responsibility for his own morning routine, traveling to school using public transportation, walking, or biking rather than relying on his mom.

##### Barry 

Barry participated in 14 PE skills coaching sessions from late February to early June. Sessions occurred in person during the school day and ranged between 15 and 60 min. During the first coaching sessions, Barry and his mother described him as withdrawn, depressed, and overwhelmed by school demands. He identified “being a good friend,” “being real,” “having a sense of humor”, “being honest”, and “helping others” as his top values. He also identified snacks, homework passes, one-on-one time with adults, and listening to music as positive reinforcers. Barry identified a weekly attendance goal, with a target of attending at least four out of five classes each week.

##### Daniel

Daniel participated in PE from early March to late May for a total of seven 60 min sessions. Daniel attended core special education classes and one elective in general education. He reported struggling with class avoidance and disengagement, often leaving classes to read alone. Daniel identified his top values as “helping his family” and “being a good friend”. He also identified food, books, and time for reading, as well as Roblox gift cards as positive reinforcers. As part of his coaching sessions, Daniel set a SMART goal related to class attendance and assignment completion in Social Studies.

#### 3.1.2. Attendance and Student Engagement Trends

##### Andy

The attendance rate data available at the time were reviewed during coaching. Baseline data (3 January–6 February 2023) showed an attendance rate of 59%. Attendance was observed at 75% during the first six weeks of PE implementation (6 February–20 March 2023) and at 69% during the remaining six weeks (3 April–15 May 2023). His SEI score was lower at post-test (2.5) than at pre-test (3.1).

Parent coaching sessions with Helen provide contextual information related to observed attendance patterns. Early sessions with Helen focused on bedtime and morning routines, use of reinforcers, and Andy’s independence for getting to school. Helen described, by session five, differences in Andy’s routines and attendance compared to earlier sessions. Upon returning from spring break, Andy met his previously identified attendance goal and added a secondary goal, marking a shift in coaching focus during this period towards practicing using reminder cards for asking for breaks and breathing techniques. Andy’s attendance was lower during subsequent weeks, especially for an added elective he did not enjoy, and fluctuated throughout the rest of the year. During this time, Helen experienced increased work stress and missed sessions, which she reported alongside challenges maintaining routines. Andy was also involved in multiple discipline incidents at school and in the community, leading to suspension. Additionally, school communication was inconsistent, including notice of early dismissal from school without parental communication, which Helen described as creating confusion around attendance and complicating her monitoring. As part of parent coaching, Helen focused on school communication concerning attendance and transition planning for high school.

##### Barry

The attendance rate data available at the time were reviewed during coaching. Barry’s baseline attendance data (16 February–17 March 2023) showed full-day attendance on 39% of days, half-day attendance on 28% of days, and absences on 33% of days. During the middle PE period (20 March–13 April 2023), full-day attendance was observed at 54%, with absences at 23%. By the final period (17 April–10 May 2023), Barry attended full days 72% of the time, with absences at 6%. Barry’s SEI scores were similar at pre- and post-test (2.4 to 2.5).

Parent sessions with Nora provided context to attendance patterns amid significant family stressors. Nora’s early sessions centered on her relationship with Barry through after-school conversations and planned activities. Following spring break, Nora began a physically demanding job that significantly limited time with her son, coinciding with interruptions in parent sessions and two major crisis events involving an accidental health scare for Barry and a school fight involving getting handcuffed and threats of legal charges. Nora and Barry described this period as traumatic. Despite these disruptions, Barry’s attendance was observed at higher rates. These observations raise questions about how student-focused coaching and value-based goal setting may relate to attendance patterns under conditions of broader systemic and situational challenges.

##### Daniel

Attendance data available at the time were reviewed during coaching. During baseline (8 March–5 April 2023), Daniel attended full days 69% of the time and half days 31% of the time. Attendance was 67% for full days and 33% for half days during the middle PE period (6 April–25 April 2023). In the final PE period (27 April–11 May 2023), full-day attendance was 92%, with half-day attendance at 8%. Daniel’s SEI score differed from pre- to post-test (2.8 to 3.2).

During the final PE period, Daniel attended full days at higher rates than earlier periods. Parent coaching sessions with Diana focused on regulating caregiving stress, creating opportunities for positive parent–child connections, and shifting attention toward attendance and effort rather than deficits. During this period, Daniel’s attendance and engagement measures were higher than earlier observations.

#### 3.1.3. Social and Behavioral Outcomes at Home and School

##### Andy

**Figure 2:**
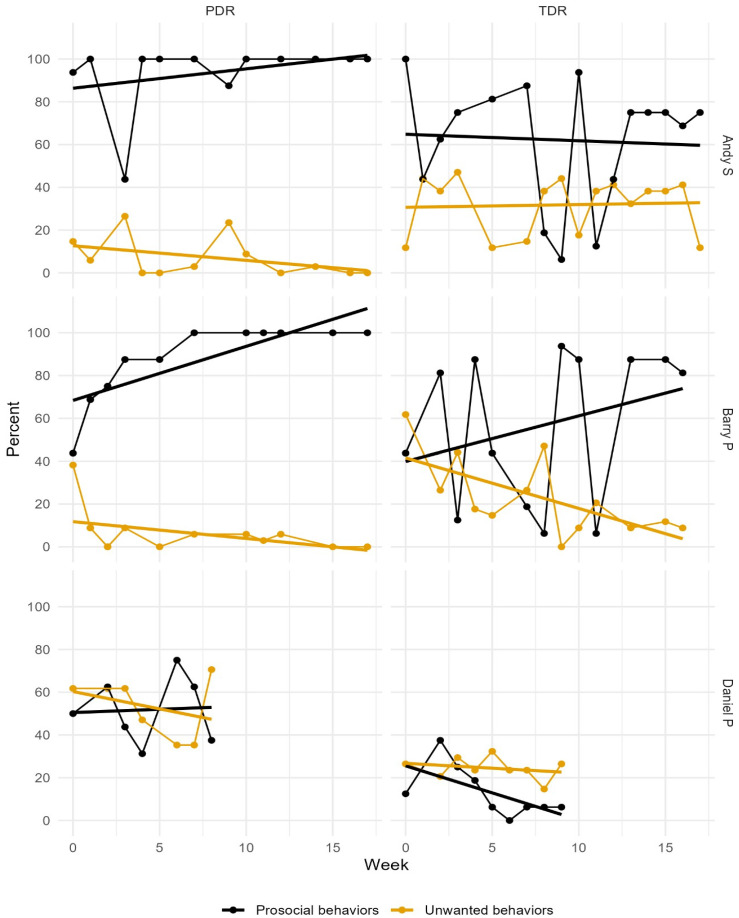
This figure displays weekly percentages of prosocial and unwanted behaviors for each student across their PE intervention period, as reported by parents (PDR; **left** column) and teachers (TDR; **right** column). The x-axis represents intervention weeks, and the y-axis represents the percentage of observed behaviors. Black lines indicate prosocial behaviors, and gold lines indicate unwanted behaviors. For each panel, solid trend lines depict the overall direction of change across weeks.

Figure 2 shows Andy’s weekly TDR and PDR ratings by his mother and special education teacher. At home, his prosocial behavior remained above 90% for most weeks, with one notable decrease following suspension, whereas unwanted behavior was lower in later weeks. School ratings showed greater variability and relatively flat trends across prosocial and unwanted behavior. On the HCSBS, Andy’s social competence remained in the at-risk range, whereas antisocial behavior positively shifted from the 97th percentile to the 48th percentile. Teacher ratings on the WMS positively shifted from the 13th to the 42nd percentile. Table 1 shows pre-post behavior rating scales for Andy. Changes in coaching focus after meeting his attendance goal during this period provide context for these behavioral patterns.

##### Barry

Figure 2 also displays Barry’s TDR and PDR ratings by his mother and special education teacher. At home, his parent-reported prosocial behavior had an initial score of 68%, with higher values in later weeks (100%), with unwanted behavior observed below 10% during the last two months of the PE period. School ratings showed variability, with higher values observed later in time. At school, teacher-reported prosocial and unwanted behavior showed less consistent patterns, but trendlines display similar directionality, with patterns reflecting lower unwanted behavior and higher prosocial behavior across the PE period. On the HCSBS, social competence scores shifted from the 23rd to the 60th percentile, consistent with increased social competence. Antisocial behavior was lower post-test (59th percentile) than pre-test (94th percentile), consistent with reduced unwanted behavior. Teacher ratings on the WMS shifted from the third to the 37th percentile. Table 1 shows pre–post behavior rating scales for Barry.

##### Daniel

Figure 2 shows TDR and PDR ratings from Daniel’s special education teacher and mother. With a shorter participation period, Daniel’s behavior ratings showed greater fluctuation. Parent reports indicated variable prosocial behavior (30–75%), with lower levels of unwanted behavior observed. School prosocial ratings remained low (6% or lower in the final five weeks), whereas unwanted behavior stayed below 30%. Daniel’s social competence scores shifted from the fourth to the sixth percentile, remaining in the high-risk range. Antisocial behavior scores were similar across pre- and post-tests (93rd–92nd percentile). Teacher-reported WMS scores shifted from the sixth to the 19th percentile, consistent with improved behavior and engagement. Table 1 shows pre–post behavior rating scales for Daniel.

### 3.2. What Descriptive Changes Are Observed in Student Engagement, Social Skills, and Behavior During PE Implementation?

**Figure 3:**
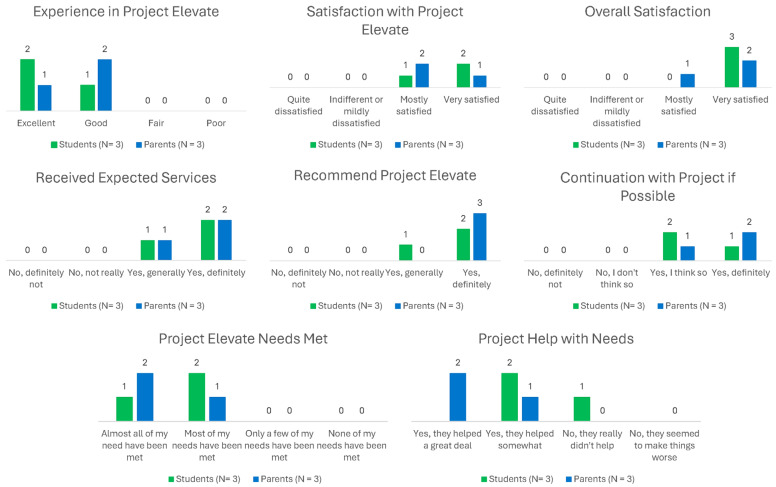
This figure presents student and parent responses to the Client Satisfaction Questionnaire (CSQ-8) and related exit interview items assessing experiences with PE. The figure displays frequency counts for student (*N* = 3) and parent (*N* = 3) ratings across eight questions: experience in PE, satisfaction with PE, overall satisfaction, receipt of expected services, recommendation of PE, willingness to continue PE if possible, extent to which PE met needs, and perceived helpfulness of the project.

To conclude PE participation, all students and parents completed the CSQ-8 and participated in exit interviews. Figure 3 presents aggregate CSQ-8 ratings, with all participants rating PE as “Good” or “Excellent.”

#### 3.2.1. Students’ Perceptions of Relevance, Practicality, and Usability

Students described PE as relevant to their school experiences and personal goals. Barry described PE as “interesting, fun, and different.” He reported that PE “actually made [him] stay in class” and improved his motivation. Daniel shared that PE “helped [him] understand why it is good to learn”, that he had “been able to talk more openly with people”, and it motivated him to work harder on homework than before. Andy shared that he “went to school more” and “stopped cussing a lot.”

When asked about skills development, Barry and Andy identified goal setting as a particularly important and useful aspect of PE. Barry stated that PE “helped me a lot with setting goals and looking at things differently when it comes to my education.” Andy shared that learning how to set goals helped him “not get into trouble” and enabled him to see what he could achieve. Daniel identified the weekly check-ins with his skills coach as the most important component of PE, explaining that his coach “is fair and listened to my problems” and “helped me understand why it is good to learn.”

#### 3.2.2. Parents’ Perceptions of Relevance, Practicality, and Benefits

Parents described PE as relevant, responsive, and practical within the context of their family needs. Helen appreciated the flexible scheduling of coaching sessions and reported that PE “gave her tools” to support improved communication with Andy and school staff. She believed the support helped her feel better equipped to advocate for her son and respond to challenges at home, adding that going through PE left them “feeling more comfortable asking for help.”

Nora described PE as especially relevant during a period marked by unemployment, language barriers, and family crises. She reported that PE provided emotional support and assistance during difficult circumstances, stating that the sessions helped her emotionally. Nora shared that PE helped her better understand Barry’s behavior and respond more effectively when he became upset, stating: “[PE] helped Barry deal with situations and it helped me to better understand Barry. Specifically, when he gets upset, how to manage his behavior. It helped us both deal with situations in a better way. Especially how to help him when he is mad.”

Diana described PE as filling a gap in existing school services by offering individualized guidance and support. She valued having structured opportunities to discuss challenges related to parenting both of her children and appreciated the practical suggestions provided through coaching. She also highlighted the value of her son having “someone to talk to and set goals for finishing middle school.”

#### 3.2.3. Perceptions of High School Preparedness

Students also reflected on how PE supported their readiness for the transition to high school. Barry reported that he did not yet feel fully prepared but believed that setting attendance and homework submission goals would help with the transition. Andy shared that he felt prepared to move on, whereas Daniel reported feeling ready to leave middle school and noted that PE helped him explain problems earlier. Nora believed that, as a result of PE, her son was somewhat more confident about school. Helen described Andy as “more mature,” with “a more positive outlook in school,” and “an easier time focusing and staying calm,” which she viewed as beneficial for his transition to high school. Diana was unsure if Daniel was ready for high school but emphasized that it was “very helpful” for her son to feel heard and important, as well as having access to one-to-one support from his coach.

## 4. Discussion

This intervention development pilot study examined descriptive student outcomes during participation in PE, a model designed to support the middle-to-high school transition for eighth grade students with SEB needs through skills coaching, parent coaching, student-led goal setting, progress monitoring, and collaboration and case management with school partners. Student and parent experiences are also presented to provide context for observed patterns. Two research questions guided this pilot study: (a) what descriptive changes were observed in student engagement, social skills, and behavior during PE implementation, and (b) how did students and parents perceive the relevance, practicality, and utility of the PE model? This discussion is organized around these questions, followed by limitations, implications for practice, and future directions.

### 4.1. Research Question 1: Observed Changes in Student Attendance, Engagement, and SEB Outcomes

One focus of PE is to empower students by working with them on self-selected goals related to academic and behavioral outcomes. Across participants, the primary student-identified goal centered on improving school attendance. Descriptive progress-monitoring data showed higher attendance rates at later timepoints compared to the baseline for each participant, an important outcome as students transition into high school and require greater independence and engagement (Jørgensen Olsen & Mehus, 2022; Zimmerman & Martinez-Pons, 1990). Prior research on student-led goal setting and student engagement highlight links among self-determined goals, student motivation, and academic and behavioral participation (Locke & Latham, 1990; Seijts & Latham, 2001). According to goal-setting theory, when students identify and define their own goals, they may demonstrate greater participation and sustained effort toward those goals, which has been associated with increased engagement in educational contexts (Rowe et al., 2017).

Preliminary results describe perceived student behavior during participation in PE within the home and school context. Parent reports indicated higher levels of prosocial behaviors and lower levels of unwanted behaviors over time across measures. At school, reported behaviors were less consistent, as reflected in the teacher-reported measures and student self-reports. For example, pre- and post-intervention teacher-reported WMS scores were higher post-test than pre-test, which is consistent with improved social behaviors. However, teacher reported TDR scores showed greater variability over time. In terms of prosocial behaviors, teacher reports varied across students, with Barry’s scores higher at later timepoints, Andy’s scores remaining similar across time, and Daniel’s scores lower at later timepoints. Similar patterns were observed for teacher-reported unwanted behaviors, with Barry’s scores lower over time, whereas Andy’s and Daniel’s scores remained largely unchanged. Student self-reported classroom engagement also varied, with Andy reporting lower scores post-test, Barry reporting similar scores across times, and Daniel reporting higher scores post-test compared to pre-test. Differences in observed outcomes across home and school contexts may be related to variability in student behavior across environments. Prior research describes associations between student behavior and contextual factors such as routines, expectations, and adult relationships, which often differ between home and school settings (Bowen et al., 2012; Garbacz et al., 2022; Hosokawa et al., 2023). At the same time, variability across context may also coincide with broader system-level conditions, in addition to individual or family factors.

Within PE, a substantial portion of support occurred through parent and skills coaching conducted outside of the classroom. Although weekly communication occurred between PE case managers and the students’ special education case managers, PE skill coaches primarily interacted with students outside the natural learning environment, and direct work on skill generalization in the moment was limited. Communication allowed for the sharing of topics and activities completed during coaching, as well as updates on the students’ action plans and strategies to be used in class when applicable. Nonetheless, variability in school-based outcomes may reflect differences in teachers’ awareness or engagement with strategies to complement and support the students’ areas of growth. Because PE skills coaches primarily worked with students outside of the classroom, opportunities for special education case managers/teachers to observe, reinforce, and integrate these strategies into classroom routines may have varied across settings, potentially contributing to inconsistent patterns in teacher-reported outcomes compared to patterns observed at home.

Greater intentionality around points of connection with school-based plans and systems may be important for implementation and for supporting the generalization of skills within classrooms. The existing literature described associations between coordinated collaboration across educational teams, and the quality of students’ educational experiences, including the implementation of support plans such as IEPs (Bambara & Kunsch, 2015; Dillon et al., 2021; Goh & Bambara, 2012; Horner et al., 2018; Mac Iver et al., 2015). Continued efforts to align skills coaching focused on student-identified goals with goals already included in their IEP may support more consistent patterns in student outcomes across contexts. In practice, PE skills coaches support students in developing and practicing skills aligned with their self-identified goals (e.g., communication, self-regulation, or goal-directed behaviors), which may at times overlap with skills addressed by school-based service providers. When alignment occurs, teachers and special education case managers may reinforce and support these skills within classroom instruction and routines. However, PE coaching may also address student-identified areas not directly targeted within existing services, positioning the PE skills coach as a complementary support rather than a substitute for school-based service delivery. In this context, integration may also involve collaboration with the PE coach and/or PE case manager in team-based planning, such as the IEP team. In these roles, PE staff may share information about the skills being practiced, describe conditions under which strategies are effective, and provide updates on student progress to support generalization across settings.

### 4.2. Research Question 2: Acceptability, Feasibility, and Utility of PE

Based on student and parent responses on the CSQ-8 and exit interviews, both groups generally rated their experience with PE positively, indicating that the model was acceptable and beneficial. Most participants reported that their needs were met and expected services were provided. Although one student reported that the project did not fully address their needs, their responses to related items suggested they felt most of their needs were met. This may reflect inconsistencies that can arise in self-reported questionnaires completed by adolescents (Fan et al., 2006). Open-ended responses similarly described positive perceptions of the model among students and parents, including appreciation for the individualized and relational nature of the intervention. Findings from student and parent reports described coaching and coordination as key implementation supports relevant to the feasibility, acceptability, and delivery of PE within school systems.

Parents largely reported that the project helped “a great deal,” whereas most students described the assistance as “somewhat” helpful. This difference may be related to parents’ perceptions of the broader scope of PE, including observations of their child, experiences with parent coaching, and the relational interactions with coaches. Parents described PE as a mechanism for supporting communication, problem solving, and emotional regulation, as well as navigating challenges related to their child’s schooling and parenting responsibilities. Parents also noted that PE was experienced as supportive during periods of heightened stress, including employment and contextual challenges.

Students described PE as helpful in concrete and task-focused ways, specifically through goal setting, regular check-ins, and having a trusted adult to talk to. Students also described regular check-ins with coaches as improving their attendance, engagement, and their understanding of school expectations. Across student and parent interviews, PE was described as improving confidence, communication, and readiness to manage upcoming demands. Two students and two parents expressed interest in continuing PE support into the summer months and extending it into high school, particularly to maintain established routines during the transition from middle to high school. This expressed interest in continued engagement suggests that students and parents perceived PE as relevant and practical during a critical period of educational transition.

Although the acceptability of PE was rated highly among students and parents, its implementation requires consideration of feasibility within typical school systems. A potential barrier to sustainability is initiative fatigue (Koh et al., 2023), particularly when new initiatives are layered onto the existing responsibilities of educators. As such, positioning PE as an approach that builds on, rather than adds to, existing MTSS and PBIS structures may enhance feasibility and scalability (August et al., 2018; Fallon et al., 2026). Within this framework, elements of PE may be integrated into existing roles and routines. For example, school psychologists or behavior specialists may support individualized planning, while teachers and other school staff may reinforce goal-setting practices within instructional contexts. School counselors or social workers may take on aspects of ongoing coaching and coordination. This distributed approach may reduce the need for additional personnel while supporting alignment with existing systems of support (Cook et al., 2019). At the same time, the model includes individualized coaching and coordination, which may require additional resources depending on school capacity. Consideration of staffing models, training requirements, and ongoing coordination efforts is therefore critical for sustainability (August et al., 2018; Cook et al., 2019; McIntosh et al., 2014). Future research is needed to identify which components of PE are most essential and to examine strategies for integrating these components within existing school systems in a way that maximizes impact while minimizing burden.

### 4.3. Limitations and Future Research

Although this intervention development pilot study offers insights into the implementation and observed outcomes of PE, several limitations should be noted. First, the case study design allowed for in depth examination of individual experiences; however, the small sample size of three students and their parents limits the generalizability of findings. As a result, conclusions from this study should be interpreted with caution, as they cannot be generalized across broader populations or varying school contexts. Moreover, the absence of a control condition limits the ability to attribute observed changes in outcomes directly to participation in PE. Duration and the nature of participation varied across student–parent dyads, with some receiving fewer coaching sessions than others, as well as variation in session duration due to scheduling and contextual factors. This variation in intervention exposure limits cross-case comparison across students. Further, follow-up data as students transitioned into ninth grade was not captured. As a result, observations related to longer-term patterns or continuity beyond middle school remain limited. Another limitation involved inconsistent access to timely attendance data before coaching sessions. At times, progress monitoring depended on school-provided data that differed in format and timing. As a result, attendance patterns could not always be described consistently across time. Lastly, reliance on teacher- or parent-reported measures introduces potential measurement limitations. Because each student was rated by a single teacher and parent across different measures, findings reflect rater-specific perceptions of student behavior over time and interpretations are limited to possible reporter-bias and within-case patterns.

Future research examining the implementation feasibility and impact of PE with a larger number of students across multiple schools would strengthen the validity of the findings and identify ways in which coordinated supports can be embedded within existing systems. As previously noted, team integration and collaboration are foundational elements of PE. Future research may examine whether the collaborative design process used to develop PE functions as an implementation support strategy that enhances feasibility, integration, and uptake across school contexts. Continued research is needed to more effectively engage PE coaches and PE case managers within IEP teams early in the transition process to support the communication and continuity of services as students enter high school. End-of-year reflection meetings conducted as part of this project offered preliminary insight into how PE staff can facilitate shared reflection and transition planning among students, families, and school personnel, highlighting the potential value of earlier and more systematic integration within the transition process. Additionally, more rigorous examination of strategies to promote skill generalization within classroom contexts would be critical for understanding school-based outcomes. Future research may also include component analysis of the PE model to examine the relative contribution, effectiveness, and impact of each individual component (e.g., skills coaching, parent coaching, goal setting, progress monitoring, and case management). Understanding which components are most beneficial, and under what conditions, would support the refinement of the model and inform more efficient and targeted implementation. Finally, testing the full scope of the PE model, including support into the nineth grade and the integration of peer mentors in place of skills coaches, would provide important insight into potential intervention effects across the secondary transition period.

### 4.4. Implications for Practice

Although multiple limitations were noted, implications for practice may also be considered based on findings from this study. PE participants described challenges related to unclear, negative, and delayed communication from schools about their children. PE coaches and case managers were at times described as facilitating communication between school personnel and families. As such, identifying more explicit “point people” within schools to support communication with parents and promote ongoing collaboration remains an important area for consideration. Although capacity continues to be a challenge in school settings, findings from this study point to the relevance of coaching for students and parents. Participants described the importance of providing parents with time to be heard and process concerns with another supportive adult, as feelings of isolation were commonly reported. From the students’ perspective, having access to a trusted adult for consistent meetings was described as helpful, particularly in relation to discussing challenges, setting goals, and planning for the future. These observations suggest potential opportunities in which students may be paired with a mentor and provided with structured opportunities, either individually or in small groups, to engage in goal setting and future-oriented planning within schools.

### 4.5. Conclusions

Students in special education with SEB needs transitioning to high school face a range of barriers, and tailored support may assist in their academic, behavioral, and social success during this period. Participants in this study faced a range of challenges, from single parenting to demanding jobs to language and citizenship barriers. Parents also described frustration in navigating school systems that largely focused on discipline and attendance, often without clear communication or adequate support. Within this context, PE was described by parents and students as providing individualized coaching experiences and opportunities for coordination and communication across home and school during a critical transition period. This evaluation of PE provides preliminary descriptive findings related to the model and highlights the need for the continued examination of coaching, coordinated service delivery, and sustained communication as factors that may be related to how transition supports are enacted within school systems. Students navigating secondary transitions may benefit from access to consistent, supportive adults who can help facilitate communication and provide relational support during critical periods in their educational journeys.

## Figures and Tables

**Table 1:** Pre–post behavioral rating scale scores across participants.

Scale	Andy S.		Barry P.		Daniel P.	
	Pre	Post	Pre	Post	Pre	Post
Student Engagement: Total Mean Score	3.1	2.5	2.4	2.5	2.8	3.2
HCSBS Social Competence: T-Score	33.0	35.0	43.0	54.0	29.0	31.0
HCSBS Antisocial Behavior: T-Score	74.0	46.0	69.0	49.0	67.0	66.0
Walker–McConnel: Total Scale Score	82.0	98.0	71.0	95.0	75.0	85.0

## Data Availability

The raw data supporting the conclusions of this article will be made available by the authors on request.

## Supplementary Materials

The following supporting information can be downloaded at: https://www.mdpi.com/article/10.3390/bs16060984/s1, Table S1: Summary of Student Coaching Session Details; Table S2: Summary of Parent Coaching Session Details.

## Abbreviations

The following abbreviations are used in this manuscript:

SEB	Social, Emotional, Behavioral
PE	Project Elevate
MTSS	Multi-tiered Systems of Supports
PBIS	Positive Behavioral Interventions and Support
PDR	Parent Daily Report
TDR	Teacher Daily Report
IEP	Individualized Education Program
HCSBS	Home and Community Social Behavior Scales
WMS	Walker McConell Scale
SEI	Student Engagement Instrument
CSQ-8	Client Satisfaction Questionnaire
