# Supplementary material for: Bridging the Gap: A Case Study of Tailored Support for Students with Social, Emotional, and Behavioral Needs During the Transition to High School
Source: Behav Sci (Basel). 2026 Jun 12;16(6):984. doi: 10.3390/bs16060984 (PMC13295868; doi:10.3390/bs16060984)
Supplement: Supplementary file 1 [file behavsci-16-00984-s001.zip › behavsci-4318171-supplementary.pdf]

Table S1

Summary of Student Coaching Session Details

Name	Sessions (*N*)	Timeframe	Session Topic	Values	Reinforcers	Goals
Andy	14	2/15/23-6/12/23	Session 1: Get to Know You (GTKU) activities, completed measures, played a game<br><br>Session 2: Reviewed goal information and brainstormed ideas for goals, GTKU activities<br><br>Session 3: Completed the values card sort, reinforcer assessment, and introduction to SMART goals<br><br>Session 4: Developed his first SMART goal around attendance<br><br>Session 5: Reviewed his goal, discussed student strengths, played a game<br><br>Session 6: Played a game and talked, wanted to work on a new goal to reduce his swearing in school and when upset	"Taking care of my family" and "being kind" as top values for him and "being a good student and peer", "not giving up", and "being physically active"	Working one-on-one with an adult on schoolwork or playing a game with an adult and listening to music were reinforcing for him.	Attending and staying at school every day until spring break. As part of the goal, Andy decided to focus on going to sleep and waking up earlier in order to arrive at school on time. Due to his mother's work schedule, Andy was in charge of getting ready on his own to be prepared for her to return and give him a ride.

	Session 7: Provided coaching on self-advocacy and played a game<br>Session 8: Worked on another goal to take a break on 3 out of 5 occasions when feeling upset and using punch card<br><br>Session 9: Talked through REST (relax, evaluate, set an intention, take action) and discussed issues at school<br><br>Session 10: Planned the student's end-of-year meeting<br><br>Session 11: Reviewed REST strategy and practiced breathing skills<br><br>Session 12: Discussed peer influences and practiced his end-of-year meeting role<br><br>Session 13: Modeled and practiced structured sharing<br><br>Session 14: Final meeting completing exit interviews	

Barry	14	2/15/23-6/1/23	Session 1: Baseline data collection, GTKU activities, discussed concerns and preferences<br><br>Session 2: Completed the values card sort, discussed some of his career interests, discussed some of his short-term goals<br><br>Session 3: Discussed persistence of staying in class when uncomfortable, began discussing goals<br><br>Session 4: Discussed a more specific focus and goal for improving attendance, including some actions to complete this goal<br><br>Session 5: Discussed addition of a goal to turn in more work during class.<br><br>Session 6: This session focused on high school and listening about concerns, fears, and hopes for next year and planning	Being a good friend, being real, have a sense of humor, being honest, helping others.	Snacks, homework passes, listening to music during class<br><br>Likes adult attention, watching videos/social media, really likes cars<br><br>Interested in possibly being a teacher (or skills coach) if not something related to social media	During the new quarter, I will be in 4 out of 5 of my classes each week<br><br>Complete 75% of assignments given across all the classes<br><br>Will do 10 minutes of work and check-in during each session<br><br>If he attends 4 of 5 of his classes, skills coach will bring a special snack

	Session 7: Barry was feeling dysregulated, practiced breathing activities<br><br>Session 8: Prioritizing classes with the learning specialist for next year, completing homework together<br><br>Session 9: Discussed coping skills, how to talk to a peer, and work completion, followed up on goal<br><br>Session 10: Discussed his ER visit and a friend that is in the hospital, needed someone to talk to<br><br>Session 11: Discussed end-of-year planning and high school transition, followed up on goal<br><br>Session 12: Discussed the altercation Barry got into at school<br><br>Session 13: Meeting with his counselor and discussing high school classes, programs, and activities, followed up on goal	

			Session 14: End-of-year planning, attending class more and turning in more work, and some celebrating			
Daniel	7	4/6/23-6/1/23	Session 1: Baseline data collection, GTKU activities<br><br>Session 2: GTKU activities, open-ended questions<br><br>Session 3: Discussed his concerns and preferences in class, completed his values cart sort<br><br>Session 4: Completed goal setting<br><br>Session 5: Follow-up on goal and end-of-year planning<br><br>Session 6: Discussing high school transition, following up on goal<br><br>Session 7: Celebration and following up on goals and future plans	Helping my family (brother especially), being a good friend,	Plain croissants, Takis and Fanta, Roblox gift cards, books, and time for reading	Turn in his Social Studies homework each week and attend Social Studies each week

Table S2

Summary of Parent Coaching Session Details

Name	Sessions (*N*)	Timeframe	Session Topic	Values	Reinforcers	Goals
Nora	14	2/15/23-6/17/23	Session 1: Baseline data, Get to Know You activities, PDR<br><br>Session 2: First parent meeting questions, discussed the 4:1 ratio, discussed information for Nora to get her driver's license<br><br>Session 3: Completed the values card sort, discussed family bonding activities<br><br>Session 4: Use values card sort as a guide for goals, areas we discussed were improving her relationship with Barry, getting to know Barry's friends' parents better and his nighttime and homework routine<br><br>Session 5: Created a smart goal around improving her relationship with Barry, implemented calendar tracking goal	Improving my relationship with my son (top value, focused on this to inform goal)		Met goal: I want to improve my relationship with my son by 1) having positive after school conversations with him 4 out of 5 days a week and 2) going on at least 2 outings with him by the end of the school year. I know I will achieve this goal when my son comes to talk to me and tells me things first. I will rate our level of trust on a scale of 1-10 (low to high). This is improvement for me because improving our relationship would make things easier for me and him. We could provide for each other and offer moral support to each other. We could

	Session 6: Checked on goal progress, discussed immigration concerns for mom (will follow-up), tried translation app services<br><br>Session 7: Checked on goal progress, also reviewed Barry's goal progress, discussed personal feelings around being pre-judged as a parent<br><br>Session 8: Zoom meeting: With spring break and Nora's new job, there has been a break in our meetings, discussed her new job and Barry going to the ER<br><br>Session 9: Followed up on goal progress, we discussed productive conversations, asking more questions (open-ended) and listening versus telling and demanding<br><br>Session 10: Phone call meeting with mom after Barry was in a fight at school.<br><br>Session 11: Immediate follow-up meeting to discuss the fight		talk about important things like our future.

at school and information from the counselor and teacher

Session 12: Follow-up on goal progress, problem-solving about her work situation, completed forms for Nora to access free immigration aid services

Session 13: Follow-up on goal progress, completed forms to move into a 2-bedroom apartment, walked to apartment office to submit the forms, check on maintenance request, the office indicates there were new procedures for Nora to follow to move into a new apartment, and within the week, Nora had completed the new procedures

Session 14: Completed our final session and exit interview, although Nora did not move into a 2 bedroom at this time; she finally moved into one in the fall, and Barry is in high school and is doing pretty good

Diana	8	4/7/23-6/1/23	Session 1: Baseline data collection, first parent meeting questions, Get to Know You Activities, PDR<br><br>Session 2: Discussing challenges with Daniel’s attendance, problem-solving the procedure to take a break and arriving early to math<br><br>Session 3: Discussed Daniel switching to math class with his learning specialist, discussed self-care activities<br><br>Session 4: Discussed high school options for Daniel next year, in school, her priorities for Daniel are math and reading, created self-care goal<br><br>Session 5: Continued to discuss high school for Daniel, discussed bonding activities with her 2 sons<br><br>Session 6: Discussed potential plans with her sons for the rest of the school year and summer, had to end session early	Self-care		Self-care after boys go to bed 2 nights a week<br><br>Meetings with parent coach were enjoyable too and mom appreciated being able to share concerns and having the parent coach listening and providing support

			Session 7: Discussed small improvements for Daniel, his math attendance has improved and he is turning in more social studies assignments, Daniel is being evaluated for Autism.<br><br>Session 8: Discussed more small victories for Daniel with attendance and completing assignments, discussed end-of-year meeting		
Helen	12	2/15/23-6/14/23	Session 1: Collected initial data and learned about context and concerns<br><br>Session 2: Completed student interview<br><br>Session 3: Completed the values card sort and discussed different goals for herself and Andy<br><br>Session 4: Developed a goal to institute a bedtime routine<br><br>Session 5: Introduced a weekly planner and planned time for Andy during spring break	Being more patient	Goal to institute a bedtime routine for her and Andy. Sticking with a plan of dinner, a walk (or quality time) and phones turned off by 8:30 pm

	Session 6: Discussed following the planner, items to advocate for at school, and Andy's step-up plan<br><br>Session 7: Helen is very stressed with work and time, pivoted to focusing on self-care, adjusting expectations for herself, and taking small moments for herself<br><br>Session 8: Implementing a text reminder system to ensure fidelity of the bedtime routine and discussed ongoing challenges with her schedule<br><br>Session 9: Discussed issues with Andy and school, reviewed the bedtime routine and discussed success with it<br><br>Session 10: Spent time planning for high school and considering different school options, including an alternative school<br><br>Session 11: Identified resources and family support to help monitor and support Andy	

Session 12: Planned summer schedule, discussed high school transition, completed exit interview
